# Understanding Susceptibility to Breast Cancer: From Risk Factors to Prevention Strategies

**DOI:** 10.3390/ijms26072993

**Published:** 2025-03-25

**Authors:** Natalia García-Sancha, Roberto Corchado-Cobos, Jesús Pérez-Losada

**Affiliations:** 1 Institute of Molecular and Cellular Biology of Cancer (IBMCC-CIC), CSIC-University of Salamanca, 37007 Salamanca, Spain; rober.corchado@usal.es (R.C.-C.); jperezlosada@usal.es (J.P.-L.); 2 Institute of Biomedical Research of Salamanca (IBSAL), 37007 Salamanca, Spain

**Keywords:** breast cancer, chemoprevention, risk factors, pregnancy, estrogens, tamoxifen, selective estrogen receptor modulators (SERMs)

## Abstract

Breast cancer is the most common malignancy among women globally, with incidence rates continuing to rise. A comprehensive understanding of its risk factors and the underlying biological mechanisms that drive tumor initiation is essential for developing effective prevention strategies. This review examines key non-modifiable risk factors, such as genetic predisposition, demographic characteristics, family history, mammographic density, and reproductive milestones, as well as modifiable risk factors like exogenous hormone exposure, obesity, diet, and physical inactivity. Importantly, reproductive history plays a dual role, providing long-term protection while temporarily increasing breast cancer risk shortly after pregnancy. Current chemoprevention strategies primarily depend on selective estrogen receptor modulators (SERMs), including tamoxifen and raloxifene, which have demonstrated efficacy in reducing the incidence of estrogen receptor-positive breast cancer but remain underutilized due to adverse effects. Emerging approaches such as aromatase inhibitors, RANKL inhibitors, progesterone antagonists, PI3K inhibitors, and immunoprevention strategies show promise for expanding preventive options. Understanding the interactions between risk factors, hormonal influences, and tumorigenesis is critical for optimizing breast cancer prevention and advancing safer, more targeted chemopreventive interventions

## 1. Introduction

Breast cancer is the most frequently diagnosed cancer and the leading cause of cancer-related mortality in women worldwide. In 2022, over 2.3 million new cases were reported, accounting for 11.6% of all cancer diagnoses, with incidence rates expected to surpass 3 million annually by 2040 [1,2]. While advances in early detection and treatment have improved survival, prevention remains a major challenge. The rising incidence of breast cancer is driven by demographic shifts, reproductive trends, and lifestyle changes, including increasing life expectancy, delayed childbearing, and the adoption of Westernized habits such as reduced physical activity and higher obesity rates [3,4,5].

**Figure 1:**
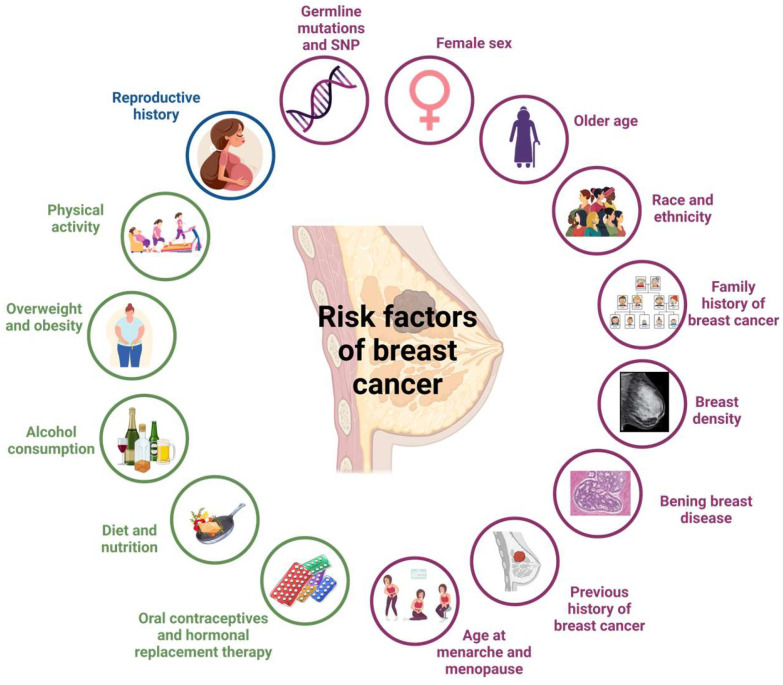
Scheme of breast cancer risk factors. Non-modifiable risk factors are highlighted in purple, modifiable risk factors in green, and reproductive history factors in blue. Created in BioRender.com. García, N. (2025) https://BioRender.com/t06q492, accessed on 23 March 2025.

Understanding breast cancer risk factors is crucial for developing effective prevention strategies. These risk factors include genetic predisposition, reproductive history, lifestyle behaviors, and hormonal influences, all of which shape an individual’s susceptibility to the disease [6,7] (Figure 1). This review summarizes key risk factors for breast cancer and discusses current and emerging chemopreventive strategies aimed at reducing its incidence.

## 2. Breast Cancer Risk Factors

### 2.1. Non-Modifiable Breast Cancer Risk Factors

#### 2.1.1. Germline Mutations and Single Nucleotide Polymorphisms

Although 5–10% of breast cancers are attributable to high-penetrance germline mutations—most notably in *BRCA1*, *BRCA2*, *TP53*, and *PTEN*—an additional 15–20% exhibit familial aggregation without a single identified high-risk gene [8]. Consequently, approximately 25–30% of breast cancers show some familial component, while the remaining cases are considered sporadic [6]. This highlights the importance of multigene panels and personalized risk assessments in clinical practice.

Germline mutations in *BRCA1* and *BRCA2*, which account for roughly 15% of hereditary breast cancer cases, compromise DNA repair mechanisms, leading to genomic instability and increased tumorigenesis [9,10,11]. Women with *BRCA1* mutations face a cumulative breast cancer risk of 44–78% and an ovarian cancer risk of 18–54% by age 70. For *BRCA2* mutation carriers, these risks are estimated at 31–56% and 2.4–19%, respectively [12,13,14,15]. Although both genes markedly increase breast and ovarian cancer risk, their associated malignancy profiles differ. *BRCA1* carriers more frequently develop triple-negative breast cancers, while *BRCA2* mutation carriers exhibit a broader tumor spectrum, including increased risks of prostate, male breast, and pancreatic cancer [14,16]. These differences underscore the importance of gene-specific risk assessment and tailored surveillance recommendations for individuals and families harboring *BRCA1* or *BRCA2* variants.

Other high-penetrance genes associated with breast cancer include *TP53*, *PTEN*, *STK11*, and *CDH1* [17,18,19,20]. Germline *TP53* mutations are linked to Li–Fraumeni syndrome, significantly increasing susceptibility to multiple tumors, such as sarcomas, adrenocortical, pancreatic, hepatocellular, and colorectal cancers. Breast cancer is the most frequent tumor in women with *TP53* mutations, with 50% of cases diagnosed before age 30 [19,21]. Women with germline *PTEN* mutations, characteristic of Cowden syndrome, have an 85% lifetime breast cancer risk. This mutation also predisposes individuals to thyroid, renal, endometrial, and skin cancers, as well as benign tumors such as hamartomas [18,22]. Germline *STK11* mutations, responsible for Peutz–Jeghers syndrome, increase breast cancer risk by up to 32% by age 60 [17,23,24]. Mutations in *CDH1*, encoding E-cadherin, cause hereditary diffuse gastric cancer and lobular breast cancer, with a 39–52% cumulative breast cancer risk by age 75 [20,25,26]. Combined with *BRCA1/2*, high-penetrance genes account for approximately 25% of hereditary breast cancers [8].

Moderate-penetrance genes, such as *ATM*, *CHEK2*, *BRIP1*, *PALB2*, *BARD1*, *MRE11A*, *NBN*, *RAD50*, *XRCC2*, *RAD51D*, and *ABRAXAS,* are involved in DNA repair and genomic maintenance [27,28,29,30,31,32]. Mutations in these genes modestly increase breast cancer risk, with carriers facing a 6–10% risk by age 60 [9]. These genes account for less than 3% of familial breast cancer cases [8].

Subsequent large-scale genome-wide association studies, including those led by the Breast Cancer Association Consortium, have significantly expanded the catalog of common susceptibility loci, with over 170 regions associated with increased breast cancer risk now listed [15,33]. The cumulative effect of these multiple low-penetrance variants can substantially modify individual risk, highlighting the polygenic nature of breast cancer predisposition [34].

While one-third of breast cancer cases show familial clustering, only 5–10% are due to high-penetrance mutations in genes such as *BRCA1* and *BRCA2* [6]. This highlights the heterogeneous genetic landscape, where moderate-risk variants, polygenic factors, and shared environmental influences may play a role [8]. Beyond high-penetrance mutations in *BRCA1*, *BRCA2*, *TP53*, and *PTEN*, current clinical practice increasingly includes the testing of moderate-risk genes such as *ATM*, *CHEK2*, and *PALB2* [35,36]. Some expanded panels also evaluate *BARD1*, *BRIP1*, *RAD51C*, and *RAD51D*, among others [8,35]. Identifying pathogenic variants in these genes guides intensive surveillance (e.g., annual MRI) and prophylactic decision-making, emphasizing the evolving role of personalized risk assessment in breast cancer prevention [37].

#### 2.1.2. Demographic Risk Factors

##### Sex

Breast cancer predominantly affects women due to hormonal influences on mammary gland development and function, but it can also occur in men, accounting for less than 1% of all cases. Men carrying *BRCA2* mutations have a significantly increased risk of developing breast cancer compared to the general male population. However, it is estimated that only about 10–15% of male breast cancer cases are associated with *BRCA2* mutations, indicating that the majority of these tumors occur without this genetic alteration [8,38]. Therefore, while *BRCA2* is a key predisposing factor in men, other genetic or environmental factors contribute to the development of male breast cancer in most cases.

##### Age

After female sex, age is the most important risk factor for breast cancer due to the accumulation of genetic mutations, epigenetic alterations and cellular senescence, so there is an increased susceptibility to tumourigenesis with increasing age [39,40]. Approximately 80% of breast cancer cases occur in women older than 50, with a median diagnosis age of 62 years [41]. The lifetime risk of developing breast cancer increases with age, reaching 2% by age 40, 3% by age 50, and over 7% by age 70 [42,43,44].

##### Race and Ethnicity

Breast cancer incidence varies significantly among ethnic groups. Non-Hispanic white and black women have the highest incidence rates, with white women reporting the highest (130.8 per 100,000), followed by black women (126.7 per 100,000). However, black women have higher incidence rates before age 45, whereas white women have higher rates between ages 60 and 84. Lower incidence rates are observed in American Indian/Alaska native, Hispanic, and Asian/Pacific islander women (94.7, 93.7, and 93.2 per 100,000, respectively) [41].

Interestingly, breast cancer incidence in Asian women who migrate to Western countries approaches that of white populations after several generations, emphasizing the role of environmental factors [45]. Regarding mortality, black women have the highest breast cancer mortality rates at all ages [41,46,47].

Numerous studies have explored factors contributing to racial disparities in breast cancer incidence and mortality. Black women are more likely to develop triple-negative, high-grade, and aggressive tumors, with younger black women experiencing double the incidence of such tumors compared to white women [48,49,50,51]. They also exhibit higher plasma levels of growth factors and estrogens, which are implicated in premenopausal breast cancer [52].

Genetic differences further contribute to these disparities. For example, a common variant at the *TERT-CLPTM1L locus*, associated with ER-negative breast cancer susceptibility, is twice as prevalent in African-American women compared to European populations [53,54]. Additionally, primary tumors in black women display a higher expression of cell cycle proteins cyclin E and P16, a higher mitotic index [55], a higher prevalence of *P53* mutations, and fewer *PIK3CA* mutations than in white women [56,57,58]. Socioeconomic factors also play a critical role. Black women are more likely to lack health insurance or to have inadequate coverage, limiting access to mammograms and influencing treatment decisions [59].

#### 2.1.3. Familial and Personal History Risk Factors

##### Family History of Breast Cancer

The risk of developing breast cancer increases with the number of first-degree relatives (mother, sister, or daughter) affected by the disease. Compared to women without affected relatives, the relative risk (RR) is 1.80, 2.93, and 3.90 for those with one, two, or three affected first-degree relatives, respectively [60,61]. This risk is higher when affected relatives are diagnosed before the age of 50 [62]. A positive family history suggests the presence of inherited genetic mutations or shared environmental exposures that predispose individuals to breast cancer.

##### Benign Breast Disease

Benign breast diseases are associated with a 70% higher risk of developing breast cancer compared to women without such conditions, although the risk varies by lesion type [63,64,65,66,67]. Histopathologically, benign lesions are classified as non-proliferative (e.g., fibrosis, simple cysts, non-sclerosing adenosis, benign phyllodes tumors, ductal ectasia, lipomas, and hamartomas), proliferative without atypia (e.g., fibroadenomas, sclerosing adenosis, radial scars, papillomatosis, and typical ductal hyperplasia), or atypical proliferative hyperplasia (e.g., atypical ductal hyperplasia and atypical lobular hyperplasia) [68]. Non-proliferative lesions confer an RR of 1.27, proliferative lesions without atypia an RR of 1.88, and atypical hyperplasias an RR of 4.24. Risk varies with the age of diagnosis: women diagnosed with atypia before age 45 have an RR of 6.99, compared to 5.02 for diagnoses between 45 and 55 years and 3.37 for diagnoses after age 55 [66]. Although benign breast disease is often regarded as a precursor state, further research is needed to understand risk factor associations among women with benign breast disease and breast cancer [69].

##### Breast Density

Breast density, defined as the percentage of epithelial and connective tissue in the breast visible on mammography, is a significant, independent risk factor for breast cancer [70,71,72,73,74,75,76,77]. High mammographic density is associated with greater susceptibility to malignant transformation due to enhanced epithelial–stromal interactions and hormonal influences [78,79]. The Breast Imaging Reporting and Data System categorizes breast density into four levels: extremely fatty (5–24%), scattered fibroglandular density (25–49%), heterogeneous density (50–74%), and extremely dense (>75%) [80,81]. An extensive meta-analysis revealed an increasing RR with higher density levels: 1.79 for 5–24% density, 2.11 for 25–49%, 2.92 for 50–74%, and 4.64 for >75% [73].

Breast density typically decreases with age, particularly during menopause. Factors such as body mass index (BMI), genetics, exogenous hormone use, diet, and reproductive history also influence breast density [82,83,84].

##### Previous History of Breast Cancer or Radiotherapy Treatment of Another Cancer

Approximately 1 in 9 women with breast cancer who survive more than a year will develop a second primary cancer, and 1 in 30 will develop contralateral breast cancer within 10 years. The risk of contralateral breast cancer is higher in women diagnosed with their first tumor before age 45, with ER-negative tumors, or those treated with radiotherapy [85,86].

Breast cancer is also one of the most common secondary tumors in survivors of childhood cancer that was treated with chest radiotherapy. For instance, childhood Hodgkin lymphoma survivors treated with radiotherapy face a 56% increased risk of breast cancer, with a median latency of 18 years [87]. This risk rises with higher radiation doses [88]. Data from Hiroshima and Nagasaki survivors confirmed the association between radiation exposure and breast cancer, showing a relative excess risk (RER) of 1.56 per Sievert, with higher risks (RER 2.65–3.94) when exposure occurred before age 20 [89,90,91].

A personal history of benign breast diseases or prior breast cancer increases the likelihood of developing subsequent malignancies due to persistent genomic alterations and residual tumor cells [92,93].

#### 2.1.4. Hormonal Risk Factors: Age at Menopause and Menarche

Early menarche and late menopause are associated with an increased risk of breast cancer due to the fact that they extend the lifetime exposure of the breast tissue to endogenous estrogens. Prolonged estrogen stimulation enhances the proliferation of mammary epithelial cells, thereby increasing the likelihood of replication errors and the accumulation of somatic mutations. In addition, sustained estrogen exposure can alter the local microenvironment by upregulating growth factors and inflammatory mediators, which further promotes genomic instability and facilitates malignant transformation. Conversely, a shorter reproductive span reduces the cumulative hormonal load and may confer a protective effect against tumorigenesis [94,95]. Epidemiological studies indicate that the age of menarche has decreased by 2–3 months per decade, from 16.5 years in 1840 to approximately 12 years today [96,97,98,99]. Factors contributing to earlier menarche include prenatal exposure to tobacco smoke and endocrine disruptors (chemicals that mimic hormone action), as well as higher BMI between ages 2 and 9 [100,101,102]. Similarly, the average age of menopause has increased, from 49 years in women born in 1910 to 50.5 years in those born in 1930 and 52.7 years in women born during the 1960s [100,103]. Factors associated with later menopause include higher educational attainment, previous oral contraceptive use, excess weight, alcohol consumption, lower physical activity, and nonsmoking status [104,105].

A systematic review of 117 epidemiological studies reported that the risk of breast cancer increases by 5% for each year earlier that menarche occurs (using 13 years as the reference). Similarly, the risk rises by 2.9% for each year menopause is delayed beyond age 51.5 [94].

Although early menarche and late menopause are recognized risk factors for breast cancer, women in these categories do not necessarily undergo more frequent clinical breast examinations, highlighting the need for targeted awareness [106]. Different reproductive and hormonal profiles may influence the optimal ages and intervals for breast screening, indicating the potential benefits of a more personalized approach [107].

### 2.2. Modified Breast Cancer Risk Factors

#### 2.2.1. Oral Contraceptives and Hormonal Replacement Therapy Consumption

Exogenous estrogens primarily come from oral contraceptives (OCs) and hormone replacement therapy (HRT) used to manage menopausal symptoms. In 2005, the IARC classified combined OCs and HRT (containing estrogens and progestogens) as group 1 carcinogens, citing sufficient evidence of their role in human cancer development. This classification was based on the increased risk of breast and cervical cancer among women using these drugs [108,109].

A 1996 meta-analysis of 54 studies reported that women using combined OCs had an RR of 1.24 for developing breast cancer, with this increased risk persisting for up to 10 years after discontinuation. However, no significant excess risk was observed 10 years or more after stopping use [110]. More recent studies confirm that this increased risk is also present with low-dose formulations of estrogens and progestogens. The relative risk ranges from 9% for less than one year of use to 46% for more than 10 years of use. Notably, the risk decreases rapidly after discontinuation [111].

HRT use is associated with a 2.3% annual increase in breast cancer risk, a level comparable to the risk associated with each additional year of delayed menopause [112]. Five years of exposure to combined estrogen–progestin therapy increases breast cancer risk by approximately 26–30%, with progestin-containing preparations being more harmful than estrogen-only formulations. The risk also rises with longer durations of use [113,114,115].

#### 2.2.2. Lifestyle Risk Factors

##### Alcohol Consumption

Alcohol intake is associated with an increased risk of breast cancer. In 2020, IARC data estimated that nearly 100,000 new breast cancer cases worldwide were attributed to alcohol consumption [116]. Women consuming 30 grams or more of alcohol daily have an RR of 1.32 for both invasive and in situ breast cancer compared to non-drinkers [117]. Even low levels of alcohol intake (10 grams/day) increase the risk by 10% [118,119].

Alcohol increases circulating estrogen levels by enhancing the aromatase-mediated conversion of androgens to estrogens and by promoting estrogen receptor transcriptional activity. Additionally, alcohol generates acetaldehyde, a toxic metabolite, and free radicals, both of which increase the susceptibility of the mammary gland to carcinogenesis [120,121,122]. Beyond the well-documented link between alcohol intake and elevated breast cancer risk, novel investigations highlight strategies for increasing risk awareness. In young adult women, exposure to targeted health warnings led to stronger intentions to reduce alcohol consumption [123].

##### Overweight and Obesity

Overweight is defined as a BMI > 25 kg/m^2^ and obesity as BMI > 30 kg/m^2^. In postmenopausal women, a higher BMI is linked to an increased breast cancer risk, whereas, in premenopausal women, a high BMI is associated with a reduced relative risk [124,125,126,127,128,129]. A meta-analysis of 2.5 million women reported that every 5 kg/m^2^ increase in BMI decreases breast cancer risk by 8% in premenopausal women but increases the risk by 12% in postmenopausal women [127]. In premenopausal women, obesity may lead to anovulatory cycles, reducing estrogen levels and mammographic density, which could lower breast cancer risk. Conversely, in postmenopausal women, obesity increases levels of estradiol, leptin, and insulin resistance, thereby raising breast cancer susceptibility [130].

##### Physical Activity

Physical activity has long been tied to reduced incidences of colon, endometrial, and premenopausal and postmenopausal breast cancer; and newly updated epidemiologic evidence now identifies inactivity as a potential contributor to as many as 15 types of malignancies [131,132,133,134,135]. In premenopausal women, physical activity may delay menarche and reduce the frequency of anovulatory cycles, thereby lowering circulating estrogen levels. In postmenopausal women, physical activity reduces adipose tissue, leptin levels, insulin resistance, and inflammatory markers such as TNF-α and IL-6, which are produced in adipose tissue. These effects collectively reduce breast cancer risk [136,137].

##### Diet and Nutrition

Recent evidence suggests that dietary intake of specific micronutrients may influence the risk of developing breast cancer, while other dietary components, such as fiber, have been studied for their potential protective effects against various cancer types. In the case of phosphorus, a cohort study in middle-aged U.S. women found that a daily intake exceeding 1800 mg was associated with an approximately 2.3-fold increased risk of breast cancer [138]. Although the results did not reach statistical significance due to the limited sample size (RR: 2.30; 95% CI: 0.94–5.61; and *p*-trend = 0.07), the study’s clinically relevant effect size, dose–response relationship, and biological plausibility support further investigation [138].

A Mendelian randomization study also identified a potential role for magnesium and phosphorus in breast cancer risk. A one-standard-deviation-higher (0.08 mmol/L) genetically predicted circulating magnesium concentration was associated with a 17% increased overall breast cancer risk and a 20% increased risk for ER+ breast cancer. Conversely, higher genetically predicted phosphorus levels were inversely associated with ER– breast cancer (OR: 0.84, 95% CI: 0.72–0.98, and *p* = 0.03), suggesting a potential protective effect that warrants further study [139].

Regarding dietary fiber, findings from the European Prospective Investigation into Cancer and Nutrition study demonstrated that doubling total fiber intake from food could reduce colorectal cancer risk by approximately 40% [140]. Although fiber’s protective role has been more extensively studied in colorectal cancer, other studies have suggested that fruit and vegetable consumption may also reduce the risk of various cancers, including breast cancer. However, the evidence for breast cancer remains inconsistent, with case–control studies showing stronger associations than prospective cohort studies [141].

For iron intake, a large Canadian cohort study of 49,654 women found no significant association between total dietary iron or heme iron intake and breast cancer risk, even among women with high alcohol consumption or those using hormone replacement therapy [142]. However, a separate study measuring elemental levels in benign breast tissue suggested a modest positive association between zinc (OR = 1.37 and *p* = 0.04), iron (OR = 1.58 and *p*-trend = 0.07), and calcium (OR = 1.46 and *p*-trend = 0.14) and subsequent breast cancer risk. Notably, the positive association between iron levels and breast cancer risk was more pronounced in postmenopausal women (OR = 2.77; 95% CI: 1.25–6.13, and *p* = 0.008), suggesting a potential hormonal influence on iron metabolism and carcinogenesis [143].

Collectively, these findings underscore the importance of further research into the role of diet and micronutrient intake—particularly phosphorus, magnesium, zinc, iron, and calcium—in breast cancer susceptibility. Large-scale prospective studies incorporating both dietary intake assessments and biological measurements of these nutrients are needed to clarify their relevance in breast cancer etiology and prevention.

### 2.3. Reproductive History and Breast Cancer

In the 18th century, Bernardino Ramazzini, regarded as the father of occupational medicine, observed that breast cancer was more common among nuns than married women, identifying for the first time the link between reproductive history and breast cancer [144]. In 1926, Janet Lane-Claypon, a pioneer of case–control studies and a key figure in epidemiology, found that breast cancer risk was higher in women who were childless, married later, or did not breastfeed. She also observed that risk decreased with an increasing number of children [145].

An early pregnancy exerts a long-term protective effect against breast cancer. Women who experience a first pregnancy before age 20 have a 50% lower lifetime risk of developing breast cancer compared to those who have never been pregnant. However, this protective effect diminishes as the age of first pregnancy increases and becomes negligible or absent when the first pregnancy occurs after age 30 [146,147]. Additionally, the number of pregnancies and breastfeeding duration enhance this long-term protective effect [148,149]. This protective effect has been observed across all ethnicities and in experimental mammalian models, such as rats and mice, using both chemical carcinogenesis models and genetically modified animals [150,151,152,153,154,155,156].

Before the long-term protective effect manifests, pregnancy is associated with a transient increase in breast cancer risk lasting up to ten years, peaking around five years postpartum. This risk is lowest in women with early pregnancies, increases with delayed first pregnancies, and is highest in women whose first pregnancy occurs after age 35 [157,158,159]. This phenomenon, termed the “dual effect” of pregnancy, reflects the transient risk increase followed by long-term protection [160].

Understanding the mechanisms underlying pregnancy’s long-term protective effect and the transient post-pregnancy risk could be critical for developing new chemoprevention strategies and preventive measures.

#### 2.3.1. Mechanisms of Post-Pregnancy Breast Cancer

Breast cancers diagnosed during the ten years following pregnancy are associated with a higher risk of metastasis and mortality compared to breast cancers diagnosed in premenopausal women outside pregnancy. This poor prognosis occurs regardless of ER status [161,162,163,164]. In contrast, breast cancer diagnosed during pregnancy is not linked to worse outcomes [165]. Early-onset breast cancer (<45 years), often referred to as young women with breast cancer, accounts for ~12% of new breast cancer diagnoses in the United States, with ~50% of these cases being diagnosed within ten years postpartum [166,167].

One potential mediator of poor outcomes in postpartum breast cancer is mammary gland involution [168]. Postpartum mammary involution begins after weaning and involves milk stasis and remodeling of the gland to its pre-pregnancy state. This process occurs in two phases: reversible and irreversible [169].

The reversible phase involves extensive cell death, primarily via lysosome-mediated cell death. Tumor necrosis factor-alpha (TNFα) induces lysosomal membrane permeabilization, releasing lysosomal contents such as proteases and cathepsins, leading to cell death. Additional mechanisms include the activation of Bax and the phosphorylation of STAT3, driven by LIF and IL-6, and the dephosphorylation of STAT5a and STAT5b, triggering apoptosis [170,171,172,173,174]. During this phase, proteins such as sulfated glycoprotein-2 (SGP-2), interleukin-1β converting enzyme (ICE), and tissue inhibitor of metalloproteinase 1 (TIMP-1) are produced at elevated levels, inducing epithelial cell apoptosis without extracellular matrix degradation. Additionally, transforming growth factor β3 (TGF-β3) and interleukin-10 (IL-10) are overexpressed, promoting TNF-related apoptosis-inducing ligand (TRAIL) and death receptor-4 (DR-4) activity, which contribute to the extensive cell death characteristic of this stage [174]. Finally, neutrophils and viable epithelial cells, acting as “non-professional phagocytes”, play a key role in clearing apoptotic cells and milk fat globules [175,176,177].

The second phase of postlactational involution, the irreversible phase, involves significant architectural changes in the mammary gland, including basement membrane remodeling, alveolar collapse, and adipocyte redifferentiation. During this phase, plasminogen (PLG) is activated to plasmin by the serine protease kallikrein (KLK1) [178]. Plasmin degrades matrix proteins such as fibrin and laminin and activates matrix metalloproteinases (MMPs), including MMP2, MMP3, and MMP9 [179]. These MMPs remodel the basement membrane by breaking down collagen in the interstitial space and induce a second wave of apoptosis in cells unresponsive to earlier death signals [169,174,177,180]. Macrophages are responsible for clearing apoptotic cells and facilitating immune cell recruitment [176]. Finally, surrounding adipocytes undergo redifferentiation [181,182,183,184].

Physiological tissue remodeling during postpartum involution shares several features with wound healing, a process known to promote tumorigenesis [185,186,187]. These features include elevated protease activity, release of extracellular matrix fragments, fibroblast activation, increased expression of the inflammatory mediator cyclooxygenase-2 (COX-2), and significant leukocytic infiltration [186,188,189,190,191,192]. This proinflammatory state during involution may create a tumor microenvironment that facilitates the malignant transformation of preneoplastic mammary cells with acquired mutations, explaining the increased risk of breast cancer within approximately ten years postpartum.

#### 2.3.2. Mechanisms of Long-Term Protection of Pregnancy Against Breast Cancer

If breast cancer does not develop within ten years after pregnancy, early pregnancy provides a long-term protective effect against the disease [146,147].

The pregnancy process itself is the primary contributor to this protection. Studies indicate that 34 weeks of pregnancy are sufficient to confer a protective effect, regardless of whether the fetus is born alive [193]. Breastfeeding further enhances protection, particularly with longer durations [194,195]. The risk of breast cancer decreases by 4.3% for every 12 months of breastfeeding [194]. However, a recent study involving over 9 million women under 55 years of age found that breastfeeding did not reduce the risk of premenopausal breast cancer [159]. This protective effect of pregnancy and breastfeeding has been observed for both ER+ and ER− tumors and extends to women with *BRCA1/2* mutations [149,196,197,198].

The molecular and pathophysiological mechanisms underlying this protective effect remain incompletely understood. However, a combination of factors—including lobular differentiation, changes in cell behavior, stromal microenvironment alterations, and gene expression changes following pregnancy and involution—likely contribute to this phenomenon [147].

A pivotal study on breast differentiation demonstrated that women with children have more differentiated lobules (type 3) in their mammary glands compared to the undifferentiated type 1 lobules found in nulliparous women [199,200]. Cells isolated from the less differentiated type 1 lobules were shown to be more susceptible to carcinogens and exhibited a higher survival capacity [201]. However, a more recent study reported no significant differences in the proportions of differentiated and undifferentiated lobules between nulliparous and multiparous women after 18 months of postlactational involution [202]. It has been proposed that type 1 lobules in women who have been pregnant may appear similar to those in nulliparous women but might originate from the regression of type 3 lobules due to age-related changes. These lobules could be functionally and molecularly distinct from the more undifferentiated and malignancy-prone type 1 lobules in nulliparous women [203].

Beyond histological changes, alterations in the fate of mammary stem/progenitor cell subpopulations after pregnancy have been documented [204]. In mice, early pregnancy reduces the number of WNT4-secreting and hormone-sensitive luminal cells (ER+ and PR+), decreasing protumorigenic Wnt signaling and enhancing differentiation-associated Notch signaling in basal stem/progenitor cells. These changes persist over time [205]. Similar results have been observed in women, where early pregnancy reduces progenitor cells, downregulates the WNT proliferative and protumorigenic pathway, and upregulates differentiation pathways such as TGF-β [206].

Additionally, the stroma of the mammary gland in women and mice after pregnancy exhibits an expansion of non-fibrillary type I collagen, which is associated with reduced stromal stiffness and a tumor-suppressive effect [207]. A 12% decrease in mammographic density following a term pregnancy has also been reported, potentially contributing to the protective effect of pregnancy [208,209].

Regarding molecular changes, Blakely et al. first reported distinct transcriptomic profiles between nulliparous and primiparous rats. Multiple rat strains shared a common transcriptional profile of 70 parity-induced genes potentially linked to resistance to breast cancer after pregnancy. Among these genes, those downregulated by parity are primarily involved in extracellular matrix remodeling and mammary gland development, while those upregulated are associated with the TGF-β pathway and immune response [155].

In female mice, basal mammary stem/progenitor cells from parous animals exhibit lower expression of WNT pathway genes (*Lgr5*, *Axin5*, *Vcan*, and *Igfbp3*), which are involved in proliferation, and in the increased expression of Notch pathway genes (*Gata3*, *Id3*, and *Dusp1*), which are implicated in differentiation in this context [205]. Similarly, in premenopausal women who have had children, genes involved in epithelial differentiation and development, such as *EGR3*, *DSC3*, *KRT5*, and *FZD8*, remain permanently upregulated after pregnancy. Some of these genes also remain overexpressed in postmenopausal women [210,211,212]. Notably, *DSC3*, a tumor suppressor commonly silenced in breast tumors, may play a role in the protective effect of parity [212].

Pregnancy also reduces circulating and mammary IGF1 levels, with a negative correlation observed between plasma IGF1 levels and the number of pregnancies [211,213,214]. High levels of IGF1 are associated with an increased risk of breast cancer [215], suggesting that an early decrease in IGF1 levels may contribute to the protective effect of pregnancy [214].

Epigenetic changes associated with pregnancy have also been described as blocking the development of premalignant lesions [216]. Transcriptomic analysis of breast tissue from postmenopausal women who had undergone an early pregnancy revealed mammary epithelial cells enriched in heterochromatin with histone H3 trimethylated at lysine 27 (H3K27me3), a transcriptionally inactive chromatin mark [217]. Hypermethylation and silencing of the genes involved in proliferation, such as *Foxa1*, *Igf1r*, *Igf1*, *Irs1*, and *Stat5b* [218,219], and hypomethylation and activation of the genes related to differentiation and tumor suppression, such as *Elf5, Cldn4,* and *Ctcfl* [220], have been observed in the breast tissue of both women and mice with early parity.

## 3. Early Mechanisms of Breast Carcinogenesis

**Figure 2:**
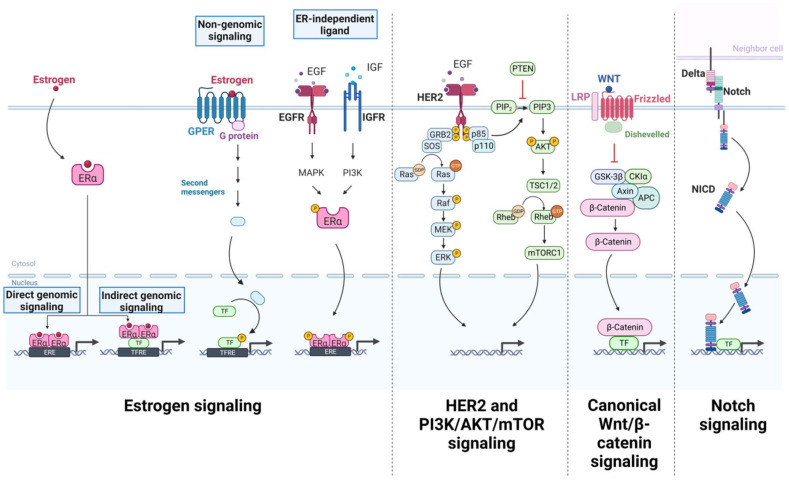
Principal signaling pathways implicated in breast carcinogenesis. Figure created with Biorender.com. García, N. (2025) https://BioRender.com/l23c841, accessed on 23 March 2025.

There are significant parallels between normal development and cancer progression at the molecular level. Many signaling pathways that regulate normal cell division are dysregulated or hijacked in cancer cells (Figure 2), a process that is driven by gene mutations and epigenetic changes, particularly in pathways related to proliferation, cell death, and differentiation [221].

### 3.1. Estrogen Signaling

The classical mechanism of estrogen signaling, known as direct genomic signaling, begins with estradiol (E2) binding to ERα or ERβ in the cytoplasm, causing receptor dimerization. This complex translocates to the nucleus, binds to estrogen response elements (EREs) in the promoters of target genes, and recruits co-activators or co-repressors. However, estradiol can also regulate genes without EREs in their promoters. In this indirect genomic pathway, E2-activated ERs interact with other transcription factors (TFs), such as activator protein-1 (AP-1) and specific protein-1 (SP-1), to modulate gene expression. Non-genomic estrogen signaling occurs when E2 binds to membrane-associated ERs, such as G protein-coupled estrogen receptor 1 (GPER1), triggering second messenger cascades. These cascades can lead to transcription factor phosphorylation and altered gene expression. Additionally, ERs can be activated in a ligand-independent manner via phosphorylation of specific residues, driven by pathways like phosphoinositide 3-kinase/protein kinase B (PI3K/AKT) and mitogen-activated protein kinase (MAPK). These pathways are activated by receptor tyrosine kinases such as the insulin-like growth factor 1 receptor (IGFR), epidermal growth factor receptor (EGFR), and human epidermal growth factor receptor 2 (HER2). Estrogens directly or indirectly influence the expression of hundreds of genes involved in processes critical to cancer, including proliferation, differentiation, survival, invasion, metastasis, and angiogenesis. Key genes regulated by estrogen include *IGF1*, *CCND1*, *NFκB*, *C/EBPβ*, *GATA1*, and *STAT5* [221,222,223,224].

Higher circulating levels of estradiol are associated with an increased risk of breast cancer [95,225,226]. Conversely, reducing estrogen exposure through oophorectomy or anti-estrogen treatments lowers breast cancer risk [227,228]. These findings have informed chemoprevention strategies aimed at controlling estrogen signaling and levels.

### 3.2. HER2 Signaling

The human epidermal growth factor receptors (EGFR, HER2, HER3, and HER4) are tyrosine kinase receptors expressed in normal tissues and various cancers. Ligand binding and subsequent receptor dimerization lead to the phosphorylation of tyrosine residues in the intracellular domain of HER2, activating downstream signaling pathways, including the MAPK and PI3K pathways [229,230]. *HER2* amplification results in protein overexpression, functioning as a potent oncogene that promotes mammary tumorigenesis both in vitro and in vivo [231,232]. *HER2* overexpression and amplification is observed in 15–20% of breast cancers [233].

### 3.3. Canonical WNT/β-Catenin Signaling

The canonical WNT/β-catenin pathway is activated when Wnt proteins bind to the co-receptors Frizzled and LRP5/6. This interaction recruits Axin and Disheveled to the plasma membrane, inhibiting glycogen synthase kinase-3β (GSK-3β). GSK-3β inhibition prevents β-catenin degradation, allowing its cytoplasmic accumulation and subsequent translocation to the nucleus. In the nucleus, β-catenin functions as a co-transcriptional activator by interacting with CREB-binding protein (CBP) and T-cell factor/lymphoid enhancing factor (TCF/LEF) transcription factors, regulating the expression of oncogenes such as *MYC* and *CCND1*, among others [234]. In breast cancer, WNT signaling is often constitutively activated. Approximately 50% of clinical breast cancer cases exhibit high levels of stabilized β-catenin, while DVL, a positive regulator of WNT signaling, is amplified in 50% of cases [235].

### 3.4. Notch Signaling

Notch signaling is initiated when ligands (Delta-like 1, 3, and 4 and Jagged 1 and 2) bind to Notch receptors, inducing enzymatic cleavage of the receptor. The Notch intracellular domain (NICD) is subsequently released and translocates to the nucleus, where it binds transcription factor CSL and co-factors to activate target gene transcription. Notch signaling promotes cell proliferation by inducing the transcription of cyclins A, B, and D1, as well as Hes/Hey family members. It also activates oncogenic pathways, including c-MYC, RAS, and WNT. Furthermore, Notch signaling is anti-apoptotic, upregulating anti-apoptotic proteins such as BCL-2, inhibiting caspases both directly and indirectly, and activating survivin. Additionally, it stimulates AKT, leading to P53 stabilization and inhibition of p21 and p15, contributing to apoptosis resistance [236].

In vivo, increased Notch activation induces mammary gland tumor formation [237], while in vitro, Notch overexpression transforms mammary epithelial cells [238]. In humans, deregulation of Notch signaling, particularly its crosstalk with WNT, is an early event in breast cancer tumorigenesis [239,240,241].

### 3.5. PI3K/AKT/mTOR Signaling

The PI3K/AKT/mTOR pathway is a central intracellular signaling network that drives cell proliferation in response to upstream stimulation by tyrosine kinase growth factor receptors, including IGF1R, EGFR, and HER2. PI3K is a heterodimer composed of a regulatory subunit (p85) and a catalytic subunit (p110). Upon activation, PI3K phosphorylates phosphatidylinositol 4,5-bisphosphate (PIP2) to phosphatidylinositol 3,4,5-triphosphate (PIP3), which, in turn, phosphorylates AKT. The tumor suppressor PTEN opposes this action by dephosphorylating PIP3 back to PIP2. Phosphorylated AKT inhibits TSC, leading to mTORC1 activation, which promotes angiogenesis and cell proliferation while inhibiting apoptosis [242].

There are three isoforms of the catalytic subunit p110—p110α (encoded by *PIK3CA*), p110β, and p110δ. The regulatory subunits, p85α, p85β, and p55γ, are encoded by *PIK3R1*, *PIK3R2*, and *PIK3R3*, respectively. Activating mutations in *PIK3CA* are found in up to 70% of proliferative benign lesions and in 35.7% of breast cancers [243]. Other alterations enhancing PI3K signaling in breast tumors include *PTEN* loss (~30%), *PIK3R1* mutations (3%), *AKT1* mutations (1.4–8%), and *HER2* amplifications [244,245].

### 3.6. Additional Pathways

Other pathways involved in breast cancer development include Sonic Hedgehog (SHH) signaling, transforming growth factor-β/small mothers against decapentaplegic (TGF-β/SMAD), nuclear factor kappa-light-chain-enhancer of activated B cells (NFκB), and Janus Kinase/Signal Transducer and Activator of Transcription (JAK/STAT) [221]. In the context of *BRCA1/2* mutations, RANK signaling plays a pivotal role in mammary carcinogenesis [246,247].

## 4. Prevention Strategies for Breast Cancer

Current trends indicate an increasing prevalence of delayed childbearing. In the USA, 18% of women giving birth in 2018 were aged 35 years or older, compared to 15% in 2013, 11% in 2002, and 8% in 1990 [248]. Additionally, a growing number of young women (aged 15–44) are being diagnosed with cancer, with post-pregnancy breast cancer accounting for half of these cases [167]. These trends underscore the urgent need to develop strategies for preventing breast cancer in general and post-pregnancy breast cancer in particular. Table 1 lists current breast cancer prevention strategies.

### 4.1. Breast Surgery

Bilateral prophylactic mastectomy is the most effective strategy for reducing breast cancer risk in women at high risk. This procedure reduces the risk by up to 95% in women with germline mutations in the *BRCA1* or *BRCA2* genes and by up to 90% in women with a strong family history of breast cancer [249,250,251,252]. However, this is a highly aggressive surgery with significant physical and psychological consequences for the patient [253].

### 4.2. Chemoprevention of Breast Cancer

The FDA has approved chemoprevention using SERMs, such as tamoxifen citrate and raloxifene hydrochloride, to lower breast cancer risk in high-risk women, although its effectiveness is not as high as that of mastectomy. Aromatase inhibitors, including exemestane and anastrozole, have shown potential in risk reduction but encounter limitations and have not yet gained FDA approval. Moreover, in recent years, various drugs have been investigated for breast cancer prevention.

### 4.3. Selective Estrogen Receptor Modulators

The approval of tamoxifen for breast cancer chemoprevention was based on findings from the National Surgical Adjuvant Breast and Bowel Project Prevention-1 (NSABP P-1) trial, the first randomized study to demonstrate tamoxifen’s efficacy in primary breast cancer prevention among high-risk women. In this trial, 13,388 women with three risk profiles (age > 60 years, age 35–59 years with a Gail model 5-year predicted breast cancer risk ≥ 1.66%, or age 35–59 years with a history of carcinoma in situ) were randomized to receive tamoxifen (20 mg/day) or a placebo for five years. After a mean follow-up of five years, tamoxifen reduced invasive breast cancer incidence by 49% and ER+ breast cancer by 69% in both premenopausal and postmenopausal women [254]. Similarly, the IBIS-I randomized controlled trial demonstrated a significant reduction in ER+ breast cancer and ductal carcinoma in situ risk with tamoxifen, with benefits persisting more than ten years after treatment cessation [228].

Raloxifene, originally approved for treating postmenopausal osteoporosis, has also shown efficacy in reducing breast cancer risk. It reduced breast cancer incidence in postmenopausal women by 72% after four years of treatment. After eight years, the reduction was 66% for invasive breast cancer overall and 76% for ER+ invasive breast cancer [255,256].

Lasofoxifene, a third-generation SERM, has shown significant promise in reducing breast cancer risk. After five years of treatment, it reduced total breast cancer (including in situ) by 79% and ER+ invasive breast cancer by 83% compared to a placebo [257]. However, it has not yet received FDA approval for chemoprevention use.

Despite their efficacy, SERMs are associated with widespread and bothersome side effects, including menopausal symptoms such as hot flashes and night sweats, leading to poor adherence; approximately 40% of women discontinue therapy [258]. Tamoxifen further increases the risk of thromboembolic events (e.g., venous thrombosis and pulmonary embolism) and endometrial cancer. In contrast, raloxifene has a lower risk of these adverse events than tamoxifen [259,260,261], but is not suitable for use in premenopausal women. Additionally, both tamoxifen and raloxifene only prevent the development of ER+ breast cancer.

### 4.4. Aromatase Inhibitors

Aromatase inhibitors have demonstrated efficacy in reducing the risk of ER+ breast cancer in postmenopausal women. Compared to a placebo, exemestane reduces the annual risk of invasive breast cancer by up to 65% [262], while anastrozole achieves a 49% reduction [263,264]. Although aromatase inhibitors have fewer side effects than SERMs, they may increase the risk of osteoporosis and bone fractures. Additionally, they are ineffective for chemoprevention in premenopausal women [264].

### 4.5. Metformin

Metformin, a first-line treatment for type 2 diabetes, activates 5′-AMP-activated protein kinase (AMPK), leading to suppression of the mammalian target of the rapamycin (mTOR) pathway and reduced tumor cell proliferation [265]. It also lowers circulating insulin levels and IGF-1 bioactivity by decreasing hepatic gluconeogenesis [266], which is relevant given the known association between elevated IGF-1 levels and increased breast cancer risk [215]. Observational studies have reported a reduced incidence of breast cancer in diabetic patients on long-term metformin therapy [267]. Ongoing clinical trials are evaluating metformin as a chemopreventive agent in high-risk women, including those with atypical hyperplasia or ductal carcinoma in situ (NCT01905046), and in overweight or obese patients to assess its effects on breast tissue density (NCT01793948). Although the exact role of metformin in breast cancer prevention requires further validation, these trials may clarify whether its metabolic and anti-proliferative actions can translate into clinically meaningful protection against breast cancer.

### 4.6. RANK/RANKL Inhibition

RANK/RANKL are essential regulators of bone remodeling. Still, they also contribute significantly to the pathogenesis of breast cancer, particularly in *BRCA1* mutation carriers [246]. RANK+ luminal progenitors in *BRCA1*-mutated breast tissue exhibit higher proliferative capacity and are considered precursors to basal-like breast cancers. Preclinical studies show that genetic or pharmacological inhibition of RANKL can decrease the formation of preneoplastic lesions, slow tumor progression, and reduce mammary tumorigenesis in Brca1-deficient mouse models [268]. Denosumab, a monoclonal antibody against RANKL, is approved for treating osteoporosis and preventing skeletal-related events in metastatic bone disease. Its potential to prevent breast cancer in high-risk *BRCA1* mutation carriers when administered biannually for five years is under investigation (NCT04711109). By blocking RANKL, denosumab may reduce proliferative signals in *BRCA1*-mutant cells, potentially offering a novel targeted chemopreventive option.

### 4.7. Progesterone Antagonists

Progesterone plays key roles in normal breast development and in certain subtypes of breast cancer, particularly those driven by *BRCA1* mutations. Mifepristone, a progesterone receptor antagonist, has been shown to inhibit Brca1-mediated tumorigenesis in mouse models [269]. However, mifepristone’s clinical applicability is limited by its side-effect profile and its primary indication for other uses.

Ulipristal acetate, a selective progesterone receptor modulator, has fewer adverse effects and has been investigated in women at high risk for breast cancer (NCT02408770). These antiprogestins may interfere with critical progesterone-driven signaling events in mammary epithelial cells, potentially slowing or preventing the clonal expansion of susceptible progenitor populations. Further research is needed to determine whether either agent, alone or in combination with other therapies, can confer durable protection against breast cancer.

### 4.8. Non-Steroidal Anti-Inflammatory Drugs (NSAIDs)

NSAIDs target inflammatory pathways implicated in tumor initiation and progression, particularly through cyclooxygenase-2 (COX-2) inhibition. Prostaglandin E2 (PGE2), a key product of COX-2 activity, can enhance cell proliferation, angiogenesis, and immune evasion in breast tissue. Aspirin use twice weekly for five years is associated with a 14% reduction in breast cancer risk, with more significant reductions observed with longer durations (26% for 10 years and 45% for 20 years) [270]. Likewise, selective COX-2 inhibitors (e.g., celecoxib) have shown a marked decrease in breast cancer incidence among women with a strong family history [271,272,273]. Additionally, accumulating evidence suggests that sustained low-dose aspirin use may confer broad chemoprotective benefits [274]. For this reason, ongoing clinical trials are evaluating low-dose aspirin for its potential anti-inflammatory and chemopreventive effects in women with benign breast disease (NCT05557877) and celecoxib in high-risk premenopausal populations (NCT00056082). While toxicity concerns—particularly gastrointestinal and cardiovascular events—must be balanced, NSAIDs remain an intriguing avenue for breast cancer chemoprevention.

### 4.9. PI3K Inhibitors

The PI3K pathway is one of breast cancer’s most frequently dysregulated signaling cascades, with activating mutations in *PIK3CA* present in up to 70% of proliferative benign lesions [243]. Alpelisib, an orally active inhibitor of the PI3K p110α subunit, has demonstrated efficacy in advanced breast cancer patients harboring *PIK3CA* mutations. Preclinical studies also indicate that alpelisib can hinder the progression from atypical hyperplasia to invasive cancer in murine models, and limit ex vivo expansion of patient-derived precancerous cells [275]. These findings suggest that PI3K inhibitors could potentially intercept early oncogenic events, preventing malignant transformation in high-risk individuals. Future clinical trials focusing on patients with known *PIK3CA* mutations in precancerous breast lesions will help determine whether targeted PI3K inhibition can become a viable chemopreventive approach.

### 4.10. Somatostatin Analogs

TheGH/IGF-1 axis is critical for normal mammary gland development and remodeling, but chronic exposure to elevated IGF-1 levels is strongly associated with increased breast cancer risk [276,277]. Somatostatin analogs, such as octreotide and pasireotide, inhibit GH release and reduce circulating IGF-1 concentrations. Preclinical data indicate that these agents can suppress mammary epithelial proliferation and delay tumor initiation [278,279]. By modulating the GH/IGF-1 axis, somatostatin analogs might counteract pro-survival and pro-proliferative signals in early mammary carcinogenesis, rendering them candidates for preventing breast cancer—particularly in individuals with elevated baseline IGF-1. Clinical trials investigating their role in chemoprevention are limited, but they remain a promising area for future research, especially given the established link between IGF-1 and mammary carcinogenesis.

### 4.11. Low-Dose Tamoxifen

To mitigate tamoxifen’s side effects, ongoing clinical trials are evaluating lower doses and alternative administration methods [282]. For instance, trials are investigating the efficacy of tamoxifen at 5 mg/day for five years (NCT01579734) or three years (NCT01357772) [280]. A randomized phase IIb study in cancer survivors previously treated with chest irradiation demonstrated that a reduced dose of 5 mg/day significantly lowered biomarkers associated with breast cancer risk while potentially offering a more favorable side-effect profile (NCT01196936) [281]. Complementing these findings, the RENAISSANCE trial (NCT06184750) is currently assessing whether short-term changes in breast density can serve as a surrogate marker for tamoxifen efficacy in premenopausal women at elevated risk. If these strategies prove successful, low-dose tamoxifen may represent an optimized chemopreventive regimen that enhances adherence and minimizes adverse events. Moreover, the topical application of afimoxifen, a tamoxifen metabolite, in women with dense breast tissue is also under evaluation (NCT03063619).

### 4.12. Natural Products-Derived Drugs

Natural product-derived compounds, notably flavonoids and resveratrol, have received substantial attention for their potential role in reducing breast cancer risk. Flavonoids, a group of polyphenolic compounds found abundantly in fruits, vegetables, teas, and wines, exhibit anti-carcinogenic properties [283]. Epidemiological studies suggest that dietary intake of flavonoids may lower tumor risk across various organs, including the breast [283,284]. Mechanistically, these compounds exert anti-proliferative effects by inhibiting signaling pathways crucial for cancer cell growth and survival [284].

Resveratrol, a natural polyphenol present in grapes, red wine, berries, and peanuts, has demonstrated potential anti-cancer activity. Preclinical work indicates that resveratrol may reduce breast cancer cell proliferation by promoting apoptosis and regulating key cell signaling cascades [285]. Furthermore, recent studies show that resveratrol can induce autophagy-dependent tumor dormancy, highlighting possible applications in both prevention and therapy [286].

Natural compounds such as flavonoids and resveratrol show potential for breast cancer prevention by modulating pathways like PI3K/AKT and NFκB. Their low toxicity and good tolerability make them attractive alternatives or complements to conventional agents, especially in women at intermediate risk [290,291]. However, as most findings to date derive from preclinical models, additional clinical research is warranted to confirm both the efficacy and safety of flavonoids and resveratrol as chemopreventive agents in breast cancer.

### 4.13. MUC1 Vaccines

Mucin-1 (MUC1) is a transmembrane glycoprotein normally expressed on the apical surface of epithelial cells. In cancer, MUC1 undergoes aberrant glycosylation, leading to the exposure of novel peptide epitopes that can be recognized by the immune system. This characteristic makes MUC1 a promising target for immunopreventive strategies. In a phase II randomized trial in the context of colorectal adenoma prevention, vaccination with a MUC1-based vaccine resulted in a 38% reduction in the incidence of new adenomatous polyps [287].

In a phase II randomized, double-blind, placebo-controlled trial involving individuals aged 40–70 with a recent diagnosis of advanced adenoma, vaccination with a MUC1-based vaccine demonstrated immunogenicity. Specifically, 25% of vaccine recipients developed a significant immune response, defined by at least a two-fold increase in MUC1-specific IgG antibodies. While the overall reduction in adenoma recurrence was not statistically significant, among those who mounted an immune response, there was a 38% absolute reduction in adenoma recurrence compared to a placebo [288].

Building on these encouraging results, a phase I clinical trial (NCT06218303) is currently underway to evaluate the safety, immunogenicity, and preliminary efficacy of a MUC1 vaccine in postmenopausal women with hormone receptor-positive ductal carcinoma in situ. As of now, data on tolerability and immune responses in this specific context are pending, and the outcomes of this trial are eagerly anticipated to determine the vaccine’s potential to prevent the progression from in situ lesions to invasive breast cancer in high-risk individuals.

### 4.14. DNA Vaccines

DNA vaccines represent a novel immunopreventive approach by delivering plasmid DNA encoding tumor-associated antigens, stimulating a targeted immune response against premalignant or early malignant cells. In breast cancer prevention—especially among individuals harboring *BRCA1/2* mutations—DNA vaccines hold promise by preemptively inducing immunity to tumor antigens. A clinical trial led by Dr. Susan Domchek at the University of Pennsylvania is currently evaluating the DNA vaccine INO-5401, alone or in combination with INO-9012, in carriers of *BRCA* mutations (NCT04367675). This phase Ib trial investigates the side effects of INO-5401 with or without INO-9012 and evaluates a new method of administering vaccines (electroporation) in adult cancer and non-cancer patients with *BRCA1* or *BRCA2* mutations. INO-5401 consists of genes that are active in human cancers (*hTERT*, *PMSA,* and *WNT1*) and are considered effective targets for the immune system of individuals who have had cancer or for those at an increased risk of developing cancer. INO-9012 provides the gene for IL12, a component of the body’s immune system. Electroporation delivers electrical pulses through an electrode placed in a tumor to enhance the ability of anti-cancer drugs to penetrate tumor cells. Administering INO-5401 and INO-9012 may boost the immune response to the vaccine. This phase I trial aims to assess the safety and immunogenicity of the vaccine.

Moreover, previous studies with DNA vaccines have shown promising results. For example, a HER2-targeted DNA vaccine trial initiated in 2011 demonstrated long-term efficacy; ten-year follow-up data published in 2023 indicated that most participating women with advanced-stage HER2-positive breast cancer remained cancer-free [289]. These findings support further development of DNA-based immunoprevention strategies.

## 5. Conclusions

Chemoprevention represents a promising strategy to reduce the incidence and mortality of breast cancer, especially in high-risk populations. Established agents like tamoxifen and raloxifene have shown efficacy in lowering risk; however, their benefits must be considered alongside side effects and their limited effectiveness against specific subtypes, particularly ER-negative disease. Aromatase inhibitors offer an additional preventive option but are limited to postmenopausal women and pose similar safety and adherence challenges. These limitations highlight the urgent need for novel, more targeted chemopreventive strategies with improved efficacy and fewer side effects.

Recent advances in understanding the complex interplay between pregnancy, postpartum involution, and long-term breast cancer risk have opened new research avenues. Targeting key signaling pathways involved in mammary tumorigenesis may provide broader protective effects. Metformin, through its metabolic and mTOR-inhibitory actions, has demonstrated potential in both experimental models and epidemiological studies. RANK/RANKL inhibition is especially relevant for *BRCA1* mutation carriers, while progesterone antagonists may assist in preventing tumor initiation in hormone-driven contexts. Additionally, NSAIDs, PI3K inhibitors, somatostatin analogs, MUC1 vaccines, and DNA vaccines target distinct molecular mechanisms of tumorigenesis, offering multifaceted approaches to primary prevention. Moreover, natural products can target multiple pathways, have fewer side effects, and may enhance the effectiveness of conventional therapies, making them promising products for breast cancer chemoprevention.

Future research should focus on identifying biomarkers that predict individual responses to chemopreventive agents, which is crucial for optimizing their clinical use. Combining these agents with complementary strategies, such as dietary changes and exercise, may further enhance their preventive potential. Moreover, as global childbearing ages continue to rise and postpartum-related breast cancers become more common, it will be essential to refine preventive interventions tailored to these demographic shifts. Ultimately, advancing personalized medicine will be pivotal in customizing chemopreventive strategies to individual patients’ genetic and molecular profiles. By leveraging our expanding knowledge of mammary gland physiology and molecular oncology, we can improve current prevention approaches, potentially leading to significant reductions in breast cancer incidence and mortality across diverse populations.

## Figures and Tables

**Table 1:** Summary of breast cancer prevention strategies.

Prevention Strategy	Mechanism of Action	Key Findings	References
Bilateral Prophylactic Mastectomy	Surgical removal of breast tissue in high-risk women	Reduces breast cancer risk by up to 95% in BRCA1/2 mutation carriers and by up to 90% in women with a strong family history	[249,250,251,252,253]
Selective Estrogen Receptor Modulators (SERMs)	Blocks estrogen receptor signaling in breast tissue	Tamoxifen reduces invasive breast cancer incidence by 49% and ER+ breast cancer by 69%; raloxifene reduces breast cancer incidence by 72% in postmenopausal women	[254,255,256,257,258,259,260,261]
Aromatase Inhibitors (AIs)	Lowers estrogen production by inhibiting aromatase enzyme	Exemestane reduces breast cancer risk by 65%; anastrozole reduces risk by 49%	[262,263,264]
Metformin	AMPK activation and IGF-1 regulation reduce tumor cell proliferation	Observational studies suggest reduced breast cancer incidence in diabetic patients on long-term therapy; clinical trials ongoing	[265,266,267]
RANK/RANKL Inhibition (Denosumab)	Blocks RANKL signaling, reducing proliferation of BRCA1-mutant cells	Preclinical models show inhibition of preneoplastic lesion formation and tumor progression; clinical trials evaluating chemopreventive effects in BRCA1 carriers	[268]
Progesterone Antagonists (Mifepristone, Ulipristal Acetate)	Blocks progesterone-driven proliferation in mammary epithelial cells	Preclinical studies suggest inhibition of BRCA1-related tumorigenesis; clinical trials evaluating feasibility	[269]
Non-Steroidal Anti-Inflammatory Drugs (NSAIDs)	COX-2 inhibition reduces prostaglandin-mediated tumor growth	Aspirin use for ≥10 years associated with 26% reduced breast cancer risk; celecoxib shows promise in high-risk women	[270,271,272,273,274]
PI3K Inhibitors (Alpelisib)	Inhibits PI3K pathway involved in early tumor development	Preclinical models show reduced progression of atypical hyperplasia; clinical trials needed	[275]
Somatostatin Analogs (Octreotide, Pasireotide)	Reduces IGF-1 signaling associated with mammary proliferation	Preclinical models indicate suppression of epithelial proliferation and delayed tumor initiation	[276,277,278,279]
Low-Dose Tamoxifen	Maintains chemopreventive effects while reducing side effects	5 mg/day significantly lowers biomarkers associated with breast cancer risk; trials ongoing	[280,281,282]
Natural Product-Derived Compounds (Flavonoids, Resveratrol)	Antioxidant and anti-inflammatory properties modulate tumorigenic pathways	Epidemiological studies suggest flavonoids may reduce breast cancer risk; resveratrol shows apoptosis-inducing effects in preclinical models	[283,284,285,286]
MUC1 Vaccines	Induces immune response against tumor-associated MUC1 epitopes	Phase II trial in colorectal adenoma shows 38% reduction in recurrence; ongoing trial in DCIS	[287,288]
DNA Vaccines	Stimulates targeted immune response against breast cancer antigens	Ongoing trials in BRCA mutation carriers; prior HER2-targeted vaccine showed long-term efficacy	[289]
